# Case management as an opportunity for healthcare: user experiences*

**DOI:** 10.15649/cuidarte.2923

**Published:** 2023-12-20

**Authors:** Lorena Chaparro-Díaz, Cindy Lorena Valbuena-Castiblanco, Sonia Carreño-Moreno, Sandra Milena Hernández-Zambrano, Ana Julia Carrillo-Algarra

**Affiliations:** 1 . Universidad Nacional de Colombia, Bogotá D.C, Colombia. Email: olchaparrod@unal.edu.co Universidad Nacional de Colombia Universidad Nacional de Colombia Bogotá D.C Colombia olchaparrod@unal.edu.co; 2 . Universidad Nacional de Colombia Bogotá. Colombia E-mail: clvalbuenac@unal.edu.co Universidad Nacional de Colombia Universidad Nacional de Colombia Bogotá Colombia clvalbuenac@unal.edu.co; 3 . Universidad Nacional de Colombia, Bogotá D.C, Colombia. Email: spcarrernom@unal.edu.co Universidad Nacional de Colombia Universidad Nacional de Colombia Bogotá D.C Colombia spcarrernom@unal.edu.co; 4 . Fundación Universitaria de Ciencias de la Salud, Bogotá D.C, Colombia. Email: smhernandez3@fucsalud.edu.co Fundación Universitaria de Ciencias de la Salud Bogotá D.C Colombia smhernandez3@fucsalud.edu.co; 5 . Fundación Universitaria de Ciencias de la Salud, Bogotá D.C, Colombia. Email: ajcarrillo@fucsalud.edu.co Fundación Universitaria de Ciencias de la Salud Bogotá D.C Colombia ajcarrillo@fucsalud.edu.co

**Keywords:** Multimorbidity, Caregivers, Family, Case Management, Qualitative Research, Multimorbilidad, Cuidadores, Familia, Manejo de Casos, Investigación Cualitativa, Multimorbidade, Cuidadores, Família, Gestao de Casos, Pesquisa Qualitativa

## Abstract

**Introduction:**

People with multimorbidity and their caregivers are beginning to be recognized as emerging subjects of health systems. In Colombia there is no differentiated approach to care for this population, as well as its health-disease process.

**Objective:**

To understand the experience of people with multimorbidity and their caregivers after receiving a case management intervention.

**Methods and materials:**

It is a qualitative study in which 33 participants among people with multimorbidity and their caregivers who received intervention with case managers were interviewed, a comparative analysis and use to tools analytics grounded theory.

**Results:**

There are 3 dimensions that are, the actors where nursing becomes relevant as a reliable source of care; the Care Meeting, as a space created within case management to maintain trust and; Results in the health system, where the need to integrate this type of outbreak into the Colombian Health Model is confirmed.

**Discussion:**

Complementary qualitative evidence data from the central study with a greater impact on the quality of care through the therapeutic relationship at home.

**Conclusion:**

The dyad requires home support for self-management of the disease based on trust, empathy, empowerment and administrative management carried out by case managers.

## Resumen

**Introducción:**

Las personas con multimorbilidad y sus cuidadores empiezan a ser reconocidos como sujetos emergentes en los sistemas de salud. En Colombia no existe un abordaje diferenciado para la atención de esta población, así como de su proceso de salud-enfermedad.

**Objetivo:**

Entender las experiencias de personas con multimorbilidad y sus cuidadores tras recibir una intervención de gestión de casos.

**Materiales y métodos:**

Estudio cualitativo en el que se entrevistó a 33 participantes con multimorbilidad y sus cuidadores que recibieron una intervención por parte de gestores de casos; se realizó un análisis comparativo y se utilizaron herramientas analíticas de la teoría fundamentada.

**Resultados:**

Existen tres dimensiones: los actores, donde la enfermería cobra relevancia como fuente confiable de cuidado; la reunión de atención o cuidado, como un espacio creado en la gestión de casos para mantener la confianza; y resultados en el sistema de salud, donde se confirma la necesidad de integrar este tipo de avance en el modelo de salud colombiano.

**Discusión:**

Los datos cualitativos complementarios del estudio central evidencian un mayor impacto en la calidad del cuidado a través de la relación terapéutica en el hogar.

**Conclusión:**

La díada requiere acompañamiento domiciliario para la autogestión de la enfermedad que esté basado en la confianza, la empatía, el empoderamiento y la gestión administrativa llevada a cabo por los gestores de casos.

## Resumo

**Introdujo:**

As pessoas com multimorbidade e seus cuidadores estao comegando a ser reconhecidos como sujeitos emergentes dos sistemas de saúde. Na Colombia, nao há uma abordagem diferenciada para o atendimento dessa populagao, bem como para seu processo saúde-doenga.

**Objetivo:**

Compreender a experiencia de pessoas com multimorbidade e seus cuidadores após receberem uma intervengao de gerenciamento de casos.

**Métodos e materiais:**

Trata-se de um estudo qualitativo no qual foram entrevistados 33 participantes entre pessoas com multimorbidade e seus cuidadores que receberam intervengao com gerentes de caso, uma análise comparativa e o uso de ferramentas analíticas da teoria fundamentada.

**Resultados:**

Existem tres dimensóes que sao: os atores onde a enfermagem se torna relevante como uma fonte confiável de cuidados; a Reuniao de Cuidados, como um espago criado dentro do gerenciamento de casos para manter a confianga e; Resultados no sistema de saúde, onde a necessidade de integrar esse tipo de surto no modelo de saúde colombiano é confirmada.

**Discussao:**

Dados complementares de evidencias qualitativas do estudo central com maior impacto na qualidade do atendimento por meio da relagao terapeutica no domicílio.

**Conclusao:**

A díade requer apoio domiciliar para o autogerenciamento da doenga com base na confianga, empatia, empoderamento e gerenciamento administrativo realizado pelos gerentes de caso.

## Introduction

Chronic diseases are a public health problem worldwide. It is with great concern that we have seen an increase in the number of cases of people with multimorbidity (PWM), which is understood as the co-occurrence of multiple chronic conditions that may be physical and/or mental. This issue has been a subject of research due to its impact on the quality of life of people living it and on health systems^1^.

Risk factors related to multimorbidity are advanced age (> 65 years), low socioeconomic status^2^, rapid disease progression, increased functional deficit, and the occurrence of multiple critical episodes and premature deaths^3^. This situation increases the level of dependence and has an impact on quality of life, mainly in the physical domain, followed by the social and psychological aspects^4^^,^^5^.

Family Caregivers (FC) of people with multimorbidity face a variety of challenges, including coordinating care from different health care professionals, scheduling appointments, managing medications, and side effects, and dealing with psychosocial issues such as high levels of psychological symptoms and limited social connectedness^6^.

The case management model is a modality for the provision of health services aimed at complex chronic patients and primary caregivers to respond to their needs and minimize the fragmentation of care, which has been proposed as a solution to this problem^7^. One of the best-known case management models is the Kaiser Permanente model^8^, which has served as a foundation for adapting them to other contexts. This model is a way of providing continuous and comprehensive care to people with chronic diseases that enhances the resolution capacity of the primary health care level. It entails having specialized and multidisciplinary primary care teams that make the necessary decisions to avoid the onset of critical conditions that require treatment in more complex health centers. Moreover, it seeks to make these groups the leaders of the care process in its entirety; therefore, benefits are focused on reducing waiting times and costs associated with the care process^8^.

For the present study, an intervention was carried out on the dyads (PWM and their FC), which lasted a year and took place between 2019 and 2020 in Bogotá, Colombia. It focused on characterization and evaluation was performed by a case management nurse (CMN) who created a care plan that could meet the dyad's needs based on the health services available and in collaboration with an interdisciplinary team.

Once the implementation stage of the intervention was completed, a qualitative approach was used to understand the experience of people with multimorbidity and their caregivers after receiving a case management intervention^9^.

## Material and Methods

This qualitative descriptive study is part of a mixed-methods study that aims to contribute to a better understanding of the phenomenon under investigation and, in this case, to expand the results of the intervention through case management. Participants met the inclusion criteria according to the type of dyad and the role they fulfilled (caregiver or patient): Type 1: 15 traditional dyads (PWM and FC); Type 2: A PWM that did not have a FC; and Type 3: A FC of a PWM living in a geriatric home. At the time of the intervention, there were two insurers of the tax regime the companies in which affiliates were found; the patients that resided in the city of Bogotá^10^. PWMs who had a care plan arranged by a CMN and in execution, with at least 3 out of 4 home visits and 2 out of 3 follow-ups; who had recently been hospitalized and were referred to the case management intervention; and who were oriented and had intact cognitive function, were included. FCs included people over the age of 18, who were oriented and had intact cognitive function, and who was a caregiver while the person with multimorbidity was in follow-up with the CMN and had received at least 3 out of 4 home visits and 2 out of 3 follow-ups. Additionally, to participate in the interview, the participants had to be oriented and cognitively intact; In the case of people with Alzheimer's or mental illness; the participation was mainly from the caregiver. Unanalyzed databases are available at Mendely data^11^.

### Data collection and processing

The participants were first contacted to confirm that they met the inclusion criteria and to inform and clarify any questions they had about the process. If participants agreed to participate in this phase, a meeting was scheduled. Informed consent was requested prior to the beginning of the interview; this consent was the initial one for the research project, which included this qualitative phase. What was done in all cases (in person and by telephone) was to verbally validate the continuity with the participation in this phase, expanding the procedure. Semi-structured interviews were conducted to get a better understanding of the care process, the role of the CMN, and the implemented care plan of the interview.

The semi-structured interview lasted an average of 26 minutes and was conducted by two researchers. The number of participants was 33 users of the case management intervention (17 patients with multimorbidity and 16 family caregivers). A total of 17 interviews were carried out (3 face-to-face and 14 by telephone) due to the health emergency caused by COVID-19. The interviews began with an initial question: I want you to tell me, how has your experience been when participating in this form of care with the implementation of nurse case managers?, which turned out that questions related to the environment were subsequently asked in the process was presented, about the role of the case manager nurse and about the care plan carried out (See Annex 1). The interviews were audio recorded and later transcribed and analyzed. Each interview was assigned a code to maintain the confidentiality and anonymity of the participants. Atlas-ti software was purchased to systematize the data obtained.

### Data analysis

The analysis took into account Charmaz's^12^ grounded theory. Initial coding was done using nominal codes derived from a word-by-word, line-by-line analysis that segmented interviews into descriptor codes. Each interview had a summary moment with a brief description of the case and a reflective moment with the authors debating the case.

The generation of nominal codes enabled the emergence of 4 analysis approaches to respond to conceptual questions, as follows: How did the case management process take place? What were the strategies for a follow-up? Who were the subjects of care (caregiver, patient, and dyad) and what was their experience in the process? and who is the case manager from the users' perspective, and what makes her different from other professionals? During each interview, theoretical memos were written to answer these questions and to be used as part of the reflection process. Subsequently, categories that were related to each other emerged to identify aspects of case management actors, the care encounter and the results they recognize to give continuity to their care process.

A validation exercise was conducted with the research team by presenting the progress of the analysis each month. In addition, the findings of the interviews with the managers were utilized to identify common points or divergences.

The Human Research Ethics Committee of Hospital San José -FUCS, through Act 9 of May 24, 2017, Series CEISH 0164-2017, approved the study, allowing registration in the Public Registry of Clinical Trials of Cuba (RPCEC). with the identifier 848-2017^13^, for which the following activities were carried out: a doctoral student (who did not participate in the intervention) was trained in conducting interviews and qualitative analysis; a researcher with qualitative experience validated each phase of analysis; validation of the analysis was carried out during reflection meetings with the researchers involved in this phase and the other researchers in the project; and comparison of recent scientific literature. The analyzed data were not returned to the participants, bearing in mind that the intervention phase had been completed by the time of the interviews, and the social commotion caused by COVID-19, which has emotionally affected people in general; however, the results were integrated into a scientific event, during which a patient and caregiver forum was held.

## Results

### Participant characteristics

Regarding demographic data, prevalence of the female sex in both the PWM and FC groups. The dyads are PWM with 80 to 89 years FC with 50 to 69 years, married, children of the patients and spouses. The PWM are low education levels as FC.

About the health status of the PWM who took part in the study. The PWM are at least 2 chronic non communicable diseases with more frequency osteoarthritis, depressive disorders, Alzheimer's disease, heart disease (ischemic and non-specific), and COPD. In FCs, the majority reported some physical alteration, with cardiovascular alterations being the most prevalent. In addition, 31.3% report having at least 2 simultaneous illnesses. Regarding information related to care FCs claimed that care from the diagnosis, are alone as FC, take care between 2 to 5 years and dedicated 18 to 24 hours a day.

### Dimensions and sub dimensions

The results of the qualitative analysis on the experience of PWM's and their caregivers after receiving a case management intervention reflect 3 dimensions that should be considered in any intervention within the Colombian health system. The dimension of the actors of the model, which involves the level of dependency on the PWM and the response capacity of the caregiver and the nurse case manager. The second dimension is the care meeting where the conditions of the intervention are detailed. Finally, the results on the transformation in navigation in the health system. The summary of each dimension is described below:

### Dimension 1: Actors of the model

This dimension includes the dependent patient vs. the autonomous patient in case management, primary caregivers that will soon be patients because they are overburdened and exhausted with paperwork, the dyad feels supported and important, the characteristics of the nurse manager that made us connect, and the importance of a relationship with other health professionals during care transitions. Next are some expressions as an actor in the process:

"Potential patient" for dealing with paperwork: *“For example, right now, I am a potential patient. I am hypertensive, and I have to deal with my anger on top of everything else. I must be here and there doing all the things that the EPS demands to deliver the medications. I have to fight for everything, including the therapies because they stop doing them whenever they feel like it.”* G1 MAHM 001They feel valued when they receive personalized attention: *‘‘...personalizedattention, care, assistance, help, service, makes you feel important. That is true, especially for elderly patients, such as my mother-in law and my father-in-law, who are no longer here.*” G1 GAC 001

### Dimension 2: Care encounters

This dimension includes moments in case management such as an initial assessment encounter that is concrete (20 to 40 minutes), which is followed by telephone encounters to schedule the home visit and face-to-face encounters (visit) (30 minutes to 2 hours). The participants stated that these encounters took place every 2 or 3 months, although there was a need for additional visits in one case. Next, the care counter is crucial to assessment and follow-up to obtain objective data to intervention with a nursing plan with education in sleeping habits, nutrition, emotional health and healthy habits, and appropriate drug use and administrative process. The dyads named the next experience as enough encounters, although some issues are pending because the intervention was different at regular attention and is necessary continuity opportunity to receive further advice, learning about care and decongestion of the health system. The dyads show the importance of the nursing role, but the problem is low autonomy capacity in Colombia. Is pending because the dyads don't review the written instructions.

Guidance for decision-making: *“I was able to talk to her (CMN) about many things I was keeping to myself, so to speak, and she advised me and told me what I should do and how to handle the situation.” G1 MAHM 025*

Additional visits: *"...She even helped me with an extra (visit). Yes, she helped me with an extra one, so that all my siblings could be there.” G1 MAHM 028*

### Dimension 3: Results

In this dimension, it is important for the administrative management or navigation through the health system to have access to appointments with specialists and communicate with their CMN WhatsApp way for new questions. Next, the dyads named recollection of the case management process with written and verbal instructions on specific care needs, it was very positive for dyads. The dad problem as experienced by dyads is a lack of autonomy and availability in the face of healthcare systems flaws.

The relationship between the CMN and the insurance is unknown: *".Regarding her and C (insurance), I would not know to what extent they are connected. I explained things to her, and she took notes and did all things necessary. That's correct, for instance! The incompetence of the IPS (institutions of higher learning) when it comes to home therapy. As an example! Poor service from P (oxygen supply). She took notes and was constantly checking in with us to see whether the problem had been solved, and we informed her.”* G1 NKS 064

More confidence in caregiving: *"Not so much as changing, no! But I feel more confident that everything that is being done is being done well.”* G1 NKS 012

Limited manager's autonomy to reduce procedures: *"Of course, leadership and power, authority. It would be good that she has the authority because she can have leadership, but if she does not have the authority to say ‘I will eliminate that procedure' ...”* G1 MAHM 001

## Discussion

The sociodemographic profile of dyads in polypathology is similar to other profiles reported in Colombia in terms of chronicity, where intervention has not necessarily been carried out. This indicates that the phenomenon of polypathology may have been around for many years in Colombia, and only with this study that focuses on polypathology is it beginning to be recognized as important to address from an innovative and relevant model such as case management^14^^,^^15^.

Other relevant findings were the high dependence levels and need for assistance in activities that allow the acquisition of habits, such as physical activity, and that they have occasionally participated in other insurance company-sponsored programs that have resulted in increased satisfaction, adherence to treatment, and improved relationships with the nursing staff. This could be associated with their need for independence since it has been found that patients want to maintain their right to decide who best meets their care needs; in a study on elder's multimorbidity the balance between safety and independence that it is possible to mitigate integrate the caregiver in plan care; and the PWN tended to prioritize the independence and the FC the safety^16^.

The present study also found that FCs have limitations in their role, such as not having other caregivers to support their work because, they have a filial obligation to assume care. For this reason, caregivers may feel alienated because caregiving duties can take up most of their time and they are unable to have a space that allows them to build support networks^17^.

Caregivers were emotionally affected, and it was found that they felt anxiety and stress when they had to leave the patients alone to attend to other responsibilities. That review concludes that is necessary more research on the FC longitudinal experiences and the influence of some characteristics such as gender, FC age, and type of relationship, on others.

Another relevant finding is that caregivers would like to have replacements or reliefs, especially during hospitalizations, because they take on additional roles such as working or being unemployed, and/or caring for several people with illnesses. According to the literature, caregivers of people with cardiovascular diseases lose up to 82% of their work productivity due to absences related to hospitalizations and presentism; therefore, there is also an economic impact^18^, which adds the fact that chronic diseases, in general, can cause more expenses to the family^19^. Consequently, caregivers need to be replaced so that they can focus on their self-care needs and engage in activities that improve their functional status and relieve stress. It is also necessary to provide them with social support to cope with the isolation they must experience as a result of their care tasks^20^.

Both PWM and FC recognize the complexity of multimorbidity at times of crisis, such as hospitalizations. According to the literature, the management of PWM is challenging due to their level of dependency, polypharmacy and, in certain cases, their cognitive impairment, which makes the presence of a permanent caregivers mandatory^21^. Due to the number and complexity of the tasks performed, caregiving may be stressful and cause illness in caregivers, resulting in the impact of multimorbidity on both parties involved and other factors such as the economic repercussions18 and the social isolation to which they are subjected^22^.

Participants identified qualities or characteristics of the CMN that facilitated the relationship with the dyad. Concerning this finding, some studies suggest that participants perceive the CMN as a friend due to the confidence that is built between them^16^, which has a favorable impact on self-management^21^.

The care needs of the dyad were also identified; according to the literature, patients and caregivers think that CMNs are skilled professionals^22^ who are very capable of responding to their needs due to their approach.

In general, there is positive interaction with the CMNs at the different levels of care. Other studies have described that continuity of care is one of the most important factors for primary care patients, along with perceived care coordination and levels of communication with providers, which are characteristics linked to nursing. Three main factors in hospital care are associated with satisfaction with nursing care: opportunity, human dimension, and safety. The first two factors are the best rated in patient satisfaction surveys^23^ and have the most impact on the quality of the relationship with the nursing staff.

Several moments of care were discovered in this study that was not restrained by time and allowed for responding to the dyad's needs. According to the literature, spending more time with the case management nurse allows patients and caregivers to perceive that they are the nurse's priority and that she is interested in their case and their concerns^21^. In this way, it is possible to strengthen the recognition of their needs and reach care agreements with the dyad.

Regarding the encounters, the participants ask to keep them because they identify valuable aspects such as the opportunity of having additional advice, learning about caring for the elderly, and decongesting the health system. Allowing the continuity of this care model would lead to continuing caring for patients with multiple diseases, extending regular follow-ups with an interdisciplinary group, and opting for the comprehensive care promulgated by the case management model.

It was also identified that the authority of the CMNs is limited, reducing the probability of giving the expected response to the needs of the dyad.

During the development of this project, the participants received booklets and written and verbal instructions that they sometimes did not consider. This type of action by the nurse manager is one of the focuses of case management: providing information, assistance, and knowledge to patients and caregivers^24^. However, as described above, patients have poor adherence to some treatments and do things based on their knowledge and criteria without considering what is recommended by health professionals in general, but having these indications helped some caregivers to have more confidence when performing care tasks.

Participants claim that the autonomy of health professionals, especially nurses, is not sufficient because nursing is not yet recognized by the Colombian health system as a relevant actor despite having the knowledge, training, and research results to support patient care decision-making, preventing nursing from leading care processes.

Another request from caregivers is the integration of relief initiatives and more frequent face-to-face meetings. The literature establishes that the need for respite or additional support in the care at the home of chronic patients is linked to allowing caregivers to have rest and recreation spaces^25^.

Lastly, the participants also stated that they face numerous administrative challenges when dealing with the health system on a daily basis. These problems continue despite attempts to reform the health system in Colombia due to the segmentation of services and multiple procedures, which cause discomfort among users^26^. Furthermore, information on processes is not standardized either and this would allow caregivers and patients to access services more easily^27^.

## Conclusions

Case management allows acknowledging the reality of the dyad at home as the routine context of care. This allows recognizing the most pressing care needs, as well as the resources available to address them. It was identified that patients over 60 years of age have multiple morbidities and that they have a high emotional burden due to the possible complications of their multiple diseases. For this reason, they make decisions about their health to maintain their independence, but they frequently do so without considering the advice of health professionals.

Regarding caregivers, it was confirmed that women are the ones who usually take on this role and are overburdened, affecting their physical and emotional well-being. Likewise, the high economic burden experienced by caregivers due to their reduced availability to be present at work during the critical moments of patients' illness was validated by the present study. For this reason, it is necessary to implement programs that support caregivers, especially during hospitalizations, and to carry out studies that describe their actual health state.

When analyzing the two actors of the dyad, it is important to recognize that the complexity of multi pathology affects them both. In this sense, a relevant piece of information found in this research is that there are cases in which the caregiver oversees two or more dependent persons at home, which may place additional stress on them and have negative consequences on their health. Therefore, more research on this phenomenon is required.

Regarding the role of nurse managers, it should be noted that specific characteristics such as empathy, responsibility, disposition, and communication positively influence the dyad relationship and facilitate the process of care provided in the context of case management, regardless of the limitations of the health system, which do not allow a more effective response to patients and caregivers.

The care encounters in this study were extensive because the nurses had unlimited availability. Consequently, it was possible to meet the needs of the dyad and help them learn about issues that were not clear to them or improve their disease-management skills. So, these encounters were significant because they made them feel accompanied and understood, increasing security in their role as caregivers and in-patient self-management.

### Strengths and limitations

In this study, patients and caregivers expressed a desire for the program to continue and be improved, allowing for the continuation of interdisciplinary treatment at home, as well as the provision of additional services to meet the caregivers' need for respite.

Participants also stated that the nurse managers provided them with brochures or written or spoken instructions, which they felt gave them more confidence to carry out their care activities, even if they did not read or follow them on a regular basis.

It is important to clarify that grounded theory tools were used for the analysis; however, the scope was not to build a substantive theory, although an outline helps to better show the identified concept that has to do with the experience of having received a case management intervention. This is mentioned in the discussion as a consideration of the scope of the results.

The significant turnover in the position of nurse manager was a limitation of this study, as it made it difficult to follow up on the care plans proposed. Another limitation was that, because of the COVID-19 pandemic, the interviews had to be conducted over the telephone, which meant that, in most cases, they could only be conducted with one member of the dyad and for a limited amount of time.
